# Hospital and Institutional News

**Published:** 1912-09-28

**Authors:** 


### HOSPITAL AND INSTITUTIONAL NEWS.

### THE CONFERENCES CONTRASTED.

The debates of the Conference of the British Hospitals Association at Birmingham last week differed from those held last year at Manchester for a very important reason. Twelve months ago the Association met, after various important meetings had been held under its auspices in London, to express publicly the attitude of the voluntary hospitals towards the newly introduced Insurance Bill. That being the case, the discussions were the preliminary steps towards the passing of certain resolutions which were eagerly awaited by the public at large, and once the resolutions had been passed debate necessarily was eclipsed for the time being. This year the Bill has become an Act, and public interest is now centred largely on the attitude of the medical profession, which will determine to a large extent the position in which the voluntary hospitals will find themselves next January. The result has been that the Conference of 1912 has been able to dispense with the passing of resolutions, a matter of such supreme importance a year ago, and the debates, like the papers, have covered a variety of topics. It is thus unnecessary, and, indeed, impossible, to summarise the different views expressed with a view to drawing a moral from them. But, on the other hand, this gives an opportunity to our readers, which necessarily did not exist last year, of seizing on the variety of controversial points that gave life to the discussions and continuing the debate in our columns. In this way those who were prevented from attending and speaking at the meetings have still an opportunity of contributing their quota of experience or criticism as the proceedings unfold in this and the succeeding issue. We cordially hope they will do so, for no hospital secretary needs reminding that it is not always those who are most silent at public meetings who have the least valuable things to say.

### RECOVERY RATES IN MENTAL HOSPITALS.

To hear Cardiff City Mental Hospital claiming a high recovery rate is no new thing for our readers, who will be interested to hear the latest remarks of Dr. Goodall, the medical superintendent, upon this important question. The report remarks that " calculated upon the number of admissions—namely, 174 (these excluding transfers from other hospitals which were already chronic), the recovery rate is 45.28 per cent. for males and 82.60 for females. The corresponding figures for all English and Welsh mental hospitals for 1910 was 30.84 and 37.41 respectively." This very high recovery rate, says Dr. Goodall, " must be considered quite abnormal, and a recovery rate of 60 per cent. has only been reached three times, while a percentage of 50 to 60 has been attained on fourteen occasions by different institutions. Such abnormally high rates in institutions compelled to receive all cases, including many quite hopeless, are, however, due mainly to some unusually favourable circumstance or combination of circumstances not under control and only in a lesser extent to the present state of knowledge and treatment." Only last week we noticed Dr. J. S. Bolton's attitude on the limited curative aspect of the mental hospitals of the present day, and the figures we quoted of the very large number of " quite hopeless " cases admitted to the West Riding Mental Hospital, Wakefield, contrast very depressingly with the exceptional recovery rate that Dr. Goodall is able to record. Want of space alone prevents us from alluding to the fascinating details of the farm and garden work and produce at the Cardiff Mental Hospital, and those who believe in the value of vegetarian diet for epileptics will note with pleasure that the vegetables produced were valued at £649.

### THE NEW CHAIRMAN OF THE BRITISH HOSPITALS ASSOCIATION.

The impetus which the Birmingham Conference, a full report of which begins in our columns this week, has given to hospital questions and to the British Hospitals Association which was responsible for the meetings makes the election of the Association's officers a matter of public concern. It gives us the greatest satisfaction to state that the newly elected Chairman of the Council is Dr. D. J. Mackintosh, M.V.O., medical superintendent of the Western Infirmary, Glasgow, who has, with great generosity, considering the claims on his time, acted hitherto as the honorary secretary of the Association. He succeeds in the chairmanship Mr. A. W. West, of St. George's Hospital, whose year of office has been an exceptionally busy one from the work and correspondence involved by the passing of the Insurance Act. The new honorary secretary is Mr. Conrad W. Thies, whose interest in the British Hospitals Association has been unflagging from the first, while Mr. Stewart Johnson has been re-elected honorary treasurer. The new batch of intending members which the annual Conferences always produce should address their applications for membership forms to Mr. Stewart Johnson at the Children's Hospital, Great Ormond Street, W.C. He will be only too glad to give any information that may be required. The Council of the British Hospitals Association has been re-elected *en bloc*, and it is hoped that they will let it be known as soon as possible where it is intended to hold the Conference next year, a matter which it was left to them to decide at the close of the Birmingham proceedings.

### THE NEW HOSPITAL AT CHISWICK.

The latest addition to the hospitals of the Metropolis is the new general hospital which will very soon be opened in the Mall, Chiswick. A cottage hospital had been opened previously in Burlington Lane, and the demands upon it were so great—no fewer than 160 in-patients having been treated in fourteen months—that it was decided to open a larger institution. The site is, æsthetically speaking, a beautiful one, and the house, which has been converted into an up-to-date hospital, is an old one, with handsome panelling still in evidence. The old coach-house has been reconstituted as an out-patient department, and now forms a unit with dressing and porter's rooms, together with a dispensary. The house, strictly speaking, is given up to the staff, who have very comfortable quarters, and the wards are connected with it by a covered-in corridor. The accommodation will provide for ten men and eight women, together with fourteen children, the latter's ward being upstairs and possessing a balcony. The theatre also is constructed and designed on modern lines, one side being of glass, and thus a full light, whether natural or artificial, is secured.

### INTOXICANTS IN INFIRMARIES.

Penalties amounting to thirty shillings have been inflicted by a London stipendiary magistrate upon a labourer who created a disturbance at the Bermondsey Infirmary, Rotherhithe, and assaulted a police constable. It appeared from the evidence called for the Bermondsey Guardians, who prosecuted, that the prisoner had attempted to carry whiskey surreptitiously into the infirmary, but was detected. Every Monday, it is stated, cases of intoxication have occurred at this infirmary, and empty bottles have been found under the patients' beds. Owing to the impossibility of searching the visitors who flock into the wards on Sundays the prevention of this lamentable state of things has proved extremely difficult. In the circumstances of the case as reported, it certainly seems to us that the magistrate, Mr. Gill, took an extremely and undesirably lenient view of the case, and the defendant may think himself lucky to escape so easily. The particular practice which he followed has been a source of occasional and recurring difficulty at many institutions, voluntary as well as rate-supported. In the end the offender gets detected, but often not before much havoc and mischief have been caused. It may be hoped that the defendant in the present case, who was himself intoxicated at the time of the *fracas*, will be deterred from any further attempt in the same direction, and that the punishment meted out to him will serve to show others similarly minded that the pastime is an expensive one sometimes.

### THE LORD MAYOR AND HOSPITAL FUNCTIONS.

Saturday last, being the Festival of St. Matthew, the Governors of the five Royal Hospitals—St. Bartholomew's, Christ's, St. Thomas's, Bridewell, and Bethlem—accompanied by the Lord Mayor and the Sheriffs, attended in State at Christ Church, Newgate Street, in accordance with the ancient custom. This year the ceremony has added interest from the fact that the Lord Mayor is a medical man, for the first time in the history of this famous office, and that this attendance in State will mark practically the last public occasion on which Sir Thomas Boor Crosby will be associated in his official capacity with hospital work before his term of office comes to an end. Both for the profession of medicine and for the voluntary hospitals, which are its schools of teaching, it has been a wonderful mayoralty. Week by week we have been able to record the hundred and one ways in which a medical Lord Mayor is able to aid the institutions and his professional colleagues. Not merely this year has taught us is it on the great hospitals days, such as Hospital Sunday, for which this year the most stirring appeal came from the pen of the Lord Mayor in our own columns, that a medical Lord Mayor can perform valuable services for his profession, but by lending the double prestige of his training and office to those medical functions at which his official position may require him to preside. That is the point of view from which this concluding hospital function may be regarded by the hospital world. But there is also another. It is, we are bold to say, not only the hospitals that have gained from this mayoralty. The office itself has received enhanced prestige from its latest representative. He has lent its influence in a wider manner than has been possible before to the causes of medicine and charity, and, as we have emphasised before, this has set a badly needed example of the part which medical men ought to play, and which they are only beginning to play, in the public and official life of the nation.

### A COTTAGE HOSPITAL'S ENTERPRISE.

As an example of enterprise on the part of a cottage hospital we think the lecture tour inaugurated by the Workpeople's Hospital Fund Committee on behalf of the Malton Cottage Hospital deserves attention. Mr. F. Wilkinson is delivering a series of illustrated lectures on "A Tour through Yorkshire," and it is the Fund's intention to make a tour of some thirty villages in the Malton district, by which the needs of the hospital may be brought to the notice of the public. Views of the hospital are included among the lantern slides, and also a likeness of Captain Clive Behrens, the chairman of the committee. The honorary secretary, Mr. S. Ridge, warmly approves the scheme, which we commend to other cottage hospitals as an example to be followed.

### THE OPENING OF THE MEDICAL SESSION.

Next week the majority of the medical schools in London and elsewhere will start work for the winter session. Among the functions and speeches to grace the occasion we note in particular the Lord Mayor's presence in the chair at a dinner of old students of St. Thomas's Hospital on October 1. Mr. H. B. Grimsdale will give an address at St. George's on "The Present Duty of the Medical Citizen," in which, we hope, he will emphasise the importance of the profession taking a more prominent part in public life. At the London Hospital the customary lecture will be delivered by Professor Wardrop Griffith. Sir Charles Wyndham, who qualified M.R.C.S. in 1857, will distribute the prizes at the Middlesex Hospital, and Dr. W. S. Lazarus-Barlow will give an address on "Genius of the Infinitely Little." Miss Jane Walker, M.D., will speak on "Common Sense" at the London School of Medicine for Women. These are among the chief attractions of October 1, an occasion when a great competition is known to exist among the lecturers as to who shall deliver the most striking address, a matter the lay newspapers seek to decide by lavish reports of these on scientific subjects likely to attract the general public.

### A STRANGE OFFER TO AN INFIRMARY STAFF.

Many strange gifts have been offered to hospitals from motives of charity, and many strange preparations and appliances are offered for sale to them by interested manufacturers, but it is not often that a man offers to sell a portion of his skin for use in grafting operations. Such, however, was the offer made to the staff of the Bradford Royal Infirmary, but as there was no case of burning or other need for the operation, the stranger's offer was declined. Destitution apparently had brought him to make the suggestion. People with unusual skulls, or other abnormalities, have been known to sell their bodies for post-mortem dissection, or to make money by exhibiting during life limbs attacked by unusual disease, but, as far as we are aware, this is the first man to think of selling his skin. It is stated that he was a journalist.

### DOCTORS' PROTEST IN MANCHESTER.

The profession in Manchester has been thrown into strong opposition by the adoption by the City Council of the Notification of Births Act of 1907, which in effect will make it compulsory for medical men to notify the births which they attend. The attack on the Council for adopting the Act is made on two grounds. In the first place, it is said that for a medical man to notify the births which he attended would be " a serious breach of professional confidence," which no Act of Parliament could possibly justify. This seems to us, on the face of it, utterly absurd and untrue. In the first place, if it is not a breach of professional confidence to compel the doctor to notify cases of infectious disease, it is not a breach of professional confidence to compel to notify births. The argument, however, on the face of it, is such a stalking-horse that we will pass on at once to consider the second ground of attack. This is that it is unjust for the State or the municipality to impose on medical men this or any other duty without a fee. With this argument we are in most complete agreement. If services are made compulsory on the profession, the profession must be paid for performing them. We think it a pity that the Manchester doctors have not boldly taken their stand on this ground alone; but if it were thought inexpedient to do so, surely a more plausible argument could have been found in place of that urging a breach of confidence.

### SANATORIUM SCENES IN DUBLIN.

The bitter feelings excited by the erection and subsequent attack on the Peamount Sanatorium again found public expression at a meeting held at Newcastle, Co. Dublin, last week to protest against the undertaking. The Rev. Walter Hurley presided, and deplored the " outrage committed at Peamount." Mr. W. S. Giltrop, R.D.C., proposed: " That the meeting protest in the strongest possible manner against the erection of a sanatorium at Peamount; strongly condemn the action of the Kildare and Kerry County Councils in taking beds in the said sanatorium, and called on those County Councils to rescind the resolutions binding them to do so, and requested the other County Councils to refuse support to the sanatorium." He said that the people of Dublin and other counties had already an institution of the kind and paid for it. In that property there were still 200 acres for extensions if required without building another in Peamount. The Peamount project had been secretly rushed through. In spite of the fact that this resolution was declared carried, it is satisfactory to note that a large section of those present realised that, however plausible these arguments might be, it was not them so much as a hysterical and exaggerated fear of infection which had really excited the protest and the outrage. It is the common knowledge of this fact which reduces Mr. Giltrop's arguments to " excuses " in the public mind.

### SARAH BERNHARDT AND THE FRENCH HOSPITAL

It is well known that Madame Sarah Bernhardt makes a point of interesting herself in the charitable activities of the various French " colonies " she visits. Here in London, undoubtedly, one of the most important interests of the French community is the excellent hospital in Shaftesbury Avenue, an institution which on more than one occasion has been honoured by a visit from the great French actress. This time Madame Bernhardt has not forgotten it. During her season at the Coliseum the proceeds of the sale of the special souvenirs which have been printed, and which give a résumé of the plots of the various plays in which she appears, will go to the funds of the French Hospital. Actors and actresses are always good friends of our hospitals, and many an institution, provincial as well as Metropolitan, can point to specific instances of help rendered by the profession in somewhat similar fashion. The appreciation of the genius of Madame Bernhardt is not confined to the French community; her fame is deservedly world-wide, and in the capital she has a wide circle of admirers and friends. What better mark of their appreciation can they give than to endow a Sarah Bernhardt bed or cot at the French Hospital? We feel sure such an attempt, if it meets with the great actress's support, will be successful in establishing a permanent memorial to her genius in an institution wherein she has always exhibited a warm interest. At any rate we commend the suggestion to the hospital authorities.

### LADY DOCTOR'S HUSBAND IMPRISONED.

As we anticipated last week, the husband of Dr. Wilks, the lady suffragette doctor of Upper Clapton, who refused to pay income tax, has been imprisoned. For as Mr. Wilks was outside the London County Council School, at which he is a master, he was arrested and taken to Brixton Prison, and as neither he nor Dr. Wilks is understood to have the slightest intention of paying the income tax Mr. Mark Wilks seems about to undergo a somewhat lengthy term of imprisonment. On Monday a march to the prison was made by the Women's Social and Political Union, but the police prevented any demonstration.

### AN UNCONSERVATIVE SURGEON.

The recent retirement of Dr. R. A. Murray, senior honorary surgeon to the Stockport Infirmary, is interesting because his long service of twenty-five years still finds him insisting on up-to-date standards, so that the latest department of the infirmary, the laboratory, owes its inception to him. As Dr. Hyde Marriott remarked, when Dr. Murray's retirement was announced, "it is only by surgery and medicine going hand in hand with pathology and bacteriology that we can obtain the best results in hospital cases." Now a quarter of a century of hospital work, and it is actually thirty-one years since Dr. Murray was house surgeon at Stockport, does not always find a man ready to welcome the latest methods and appliances. Indeed, a case came before us only a week or two ago in which a laboratory had actually been built, but its equipment and working were being impeded through the conservative tendencies of a senior member of the staff. That is a very different state of things from the one recorded of Dr. Murray of Stockport, and we think everyone will feel that the praise and gratitude given to Dr. Murray on his retirement were as nothing compared to the statement we have quoted, that he was the father of the latest department established in the institution. To have that said of a man at the end of his services on the staff is the best proof of a fine record.

### INSTITUTIONAL SIGN-POSTS.

At the recent annual meeting of the Gloucester Dispensary Mr. George Aldridge, deputy chairman of the committee, received the thanks of an enthusiastic meeting for the efforts which he had made to increase the subscription list. During the past year the total amount received was practically doubled, and as the year began with the issue of an appeal by Mr. Aldridge, he and the committee certainly deserve praise. This is worth recording, as the most difficult appeal nowadays to launch successfully is, perhaps, an appeal connected with provident or dispensary work. No one knows this better than the man or woman who has been long interested in dispensary work. It is sometimes forgotten that the small institutions illustrate more directly the tendencies of the times than their greater contemporaries, but in reality the small institution often carries the sign-post which is found to point the way for the generality of hospitals and dispensaries a little later.

### SANATORIUM BENEFIT IN LONDON.

The machinery for providing sanatorium benefit for insured persons in London is announced by the Clerk to the London County Council to be now in working order. It is understood that sixty applications have been received, of which about half has been dealt with. It is claimed that among them are several cases which, in spite of voluntary agencies, would probably not have been dealt with until the incipient stage was passed. The London Committee has appointed a temporary sanatorium sub-committee for the cities of London and Westminster and for each Metropolitan borough. After inquiries and examination the committee, acting on the advice of their expert, decides on the treatment; if institutional the committee makes arrangements, if domiciliary or at the dispensary the local sub-committee. On every such sub-committee, thanks to the co-operation of the Metropolitan borough councils, the medical officer of health invariably serves. The sub-committees, which are allowed to meet in the borough buildings, form a link with the central organisation, and, it is hoped, will be the nucleus of the district committees which shortly will have to be appointed under the Act. A staff is being engaged, central offices are being looked for, and meanwhile applicants for sanatorium benefit can obtain the necessary forms from the acting clerk of the London Insurance Committee, County Hall, Spring Gardens, S.W., or from the respective town halls.

### THE MAYOR OF PORTSMOUTH'S ROUND-TABLE.

To save the Royal Portsmouth Hospital from municipalisation the Mayor, Sir Scott Foster, held a round-table Conference last week at which neighbouring Urban District and Parish Councils were largely represented, the members ultimately agreeing to use their persuasive powers on their district colleagues to gain increased support from the many localities which send patients freely to the institution, but subscribe with a much less lavish hand. The Chairman of the Hospital Board, Councillor Harold Pink, and the Secretary, Mr. B. Wagstaff, represented the hospital. Councillor Pink remarked that the Urban and Rural Councils had become large employers of labour and should support the hospital in the same way as private employers. If each of the outlying districts, such as Havant or Gosport, had to provide its own hospital, he pointed out that it would cost them far more and a much higher contribution than they gave at present. These arguments have common sense and logic behind them, and we hope that the Conference will be followed up by a strong campaign in the outlying districts, each of which should be presented with a table showing the roll of patients it has sent to the hospital during the past twelve months, while side by side with that should be placed the amount of its subscription. In this way the responsibility can be brought home to each district, and those which are niggardly will have their exploitation of the hospital laid bare. Such tables could not fail to be recognised as just claims by the Local Government Board, on which and on the auditor the blame was laid by some of the speakers.

### THE RECONSTRUCTION OF CHESTER INFIRMARY.

On Thursday last week the Duchess of Westminster proceeded to lay the memorial stone of the new wings which are being added to Chester Infirmary in memory of King Edward. The arrangements included a view of the infirmary and the extension, the conditions and need for which have been outlined so often in our columns. Last January the decision was arrived at upon the gift of £12,500 from Mr. Albert Wood, of Conway. The total cost has been estimated at £40,000, of which about three-quarters has been already subscribed. The architects responsible for the work are Mr. W. T. Lockwood, F.R.I.B.A., and Mr. Paul Waterhouse, F.R.I.B.A. The scheme of reconstruction does not involve the abandonment of the original buildings, 150 years old though they are; and the sections in which it will be undertaken have been allotted to various benefactors, so that Mr. Wood's gift will be devoted to the Albert Wood Wards, and the pageant funds of 1910 will pay for another distinct section of the work. The principal alterations include the removal of the kitchen to the central position now occupied by the out-patients' department, which henceforth will be much extended and transferred to the Humberstone wing. The Albert Wood Wards will consist of six or ten beds apiece. A new theatre unit will also be built. Six wards for observation or isolation cases, each with three beds, will also form part of the scheme; a new storey is to be added to the nurses' home for the additional staff which the increased accommodation for patients will render necessary. Two special wards and an operation room for eye cases are to be formed over the chapel; and the existing wards are being renovated. The chief alteration to the old building is perhaps the removal of one storey of the central block. We are glad to give these details, as from the first we have recorded the various developments of the scheme. The unsatisfactory condition of the hospital was pointed out by Sir Henry Burdett when he visited the institution in the autumn of 1909.

### MISLEADING STATISTICS.

Deaths under anæsthetics have been the cause of so much misapprehension and distorted comment that it is refreshing to turn to one side of the question which was raised in the City Coroner's Court the other day. After a verdict of "Death by misadventure" had been returned at an inquest concerning the death of a young woman patient at St. Bartholomew's Hospital, the Coroner remarked that various authorities gave widely differing proportions of deaths under anæsthetics, and the figures of the Registrar-General were not only perfectly useless from a statistical point of view, but positively misleading. It is true, of course, that many deaths under anæsthetics which occur in private houses or in nursing homes are not reported at all. Legislation is wanted, but the Government is too busy with controversial problems for any hopes to be entertained of the question being dealt with in Parliament at present.

### NEWCASTLE HOSPITAL SUNDAY FUND.

The methods of distribution adopted by the Newcastle Hospital Sunday Fund for a long time have excited contentious opinions, with the result that a special sub-committee was appointed to examine and report. It has been unanimous in deciding that no change should be made in the present system of distributing the fund without consulting the wishes of the subscribers. At the annual meeting last week the question again was brought forward, and a correspondence between the Rev. J. H. Maconachie, a member of the fund, and Mr. Alfred Appleby, its honorary secretary, has been laid before the subscribers. The gist of the matter is the allegation that those medical charities which issue letters of admission have been obtaining some additional benefit from the fund which the charities which do not issue letters do not receive, an allegation which led to the resignation of Alderman Richard Henry Holmes, for a long time the fund's honorary secretary and one of its founders. The committee of investigation found, however, that the allegation that institutions were obtaining large benefits from the sale of letters to the fund was incorrect. Mr. Appleby pointed out that the amount these institutions receive does not make up to them the loss they suffer from supplying letters to the fund. The meeting decided almost unanimously to adopt the report, and it is therefore much to be hoped that the friction which has hampered the fund's efforts will now be at an end.

### WHOOPING-COUGH AS A NOTIFIABLE DISEASE.

The Lambeth Borough Council propose making whooping-cough a compulsorily notifiable disease under the Public Health (London) Act, 1891, for a period of five years. The annual deaths from whooping-cough in Lambeth were for the two decennia 1891-1900 and 1901-10 143 and 91 respectively, the annual morbidity from the disease being in the same proportion. Dr. Priestley, the medical officer of health, is of opinion that there would be a great saving of life and ill-health amongst infants and children if notification of the disease were made compulsory.

### DR. MORRISON'S RETURN TO CHINA.

Dr. Morrison, the political adviser to the President of the Chinese Republic, has started for Peking with Mrs. Morrison, his short holiday in this country having been chiefly undertaken for the purpose of his marriage. Two stops will be made, at Paris and Berlin, and Peking will be reached in about seventeen days from the date of departure. As we stated, when his political appointment was announced, Dr. Morrison's public activities abroad and Sir Thomas Crosby's as Lord Mayor at home have given this year to the profession two striking examples of medical men in public life, which perhaps point the way to increased extra-professional activity in the profession generally.

### COTTAGE HOSPITALS AND THE ROYAL FAMILY.

It was very appropriate that at the ninth annual meeting of the Frank James Memorial Cottage Hospital, East Cowes, when the death of one of the founders, Mr. William James, had to be recorded, the President of the institution, Princess Henry of Battenberg, was able to preside. Nothing perhaps could better typify the sincerity of the Royal Family's interest in voluntary hospitals, and in particular Princess Henry's in those of the Isle of Wight. Dr. Ewen, the honorary secretary, received the President at the Town Hall, where Mr. George Groves, vice-chairman, and Mr. George Shedden, honorary treasurer, were in attendance. This cottage hospital is interesting because its beds include six for typhoid patients and because paying patients are also received. His Majesty is one of the subscribers, and probably there is no cottage hospital which has received more frequent proofs of the Royal interest.

### THE ÆSTHETIC MEMORIAL.

A portrait of the late Mr. John Powell, who had been for fifteen years a member of the committee of West Bromwich Hospital Saturday Committee, has been unveiled by the Mayor in the out-patient department of the institution. Mr. Hancock, the hospital's secretary, accepted the portrait on behalf of the committee, which realises no doubt how much the all-important local interest is fostered and transmuted into a living tradition by the possession of such memorials as these, which are visible witnesses of the personal service that alone carries on the voluntary system successfully. It is sometimes said that money is better spent on some "practical" purpose than on any kind of æsthetic memorial. We do not endorse that view, believing rather that the practical purpose gains enormously if, as in the joint scheme for the memorial to Florence Nightingale, for instance, it be accompanied by a statue or a picture, and should gladly sacrifice a part of the funds subscribed for such a purpose.

### A HOSPITAL MEMORIAL TO LISTER.

Particulars are now forthcoming concerning the memorial to Lord Lister which University College has decided to establish. A special committee has been formed, of which the Duke of Bedford is president, as he is also president of the hospital, and Sir John Tweedy is the honorary treasurer of the fund. The aim of the committee is for a local memorial to be established, of what kind will depend upon the amount of subscriptions received, but in any case a bust or a tablet will probably be placed in both the hospital and the college. Only those connected with University College or the hospital are being asked to subscribe, and these will need little reminding that Lister entered the college as an art student in 1843 and graduated Bachelor of Arts in 1847. The project is an admirable one, but why has it been put forward so comparatively late in the day? Its chances of immediate success are not increased by this period of waiting.

### THE LATE LORD LLANGATTOCK.

The Monmouth General Hospital and Dispensary has to mourn the loss of its president this week through the death of Lord Llangattock at the age of seventy-five. He was prominent in many fields, as a breeder of Shire horses, as a master of hounds, as a restorer of churches, and, finally, as a keen supporter of hospital work and voluntary charity in many fields. Monmouth has to thank him for many benefactions. This enterprise, it is worth adding, led to the tragic death of his son, the Hon. C. S. Rolls, who, as a pioneer motorist and the first airman to fly across the Channel, was killed in an aeroplane accident at Bournemouth. The Monmouth Hospital owes so much to his interest and support that it is not improbable a memorial will be raised to his memory.

### AN ENGLISH DOCTOR AND FRENCH REGULATIONS.

A rather curious case has arisen in France through the action of the house surgeon at the English hospital at Levallois, who was not qualified to practise in France, performing a post mortem on a groom who had died subsequently to laparotomy. When the local commissary of police arrived with the police doctor he refused to deliver the burial permit on the ground that the post mortem had been made by a foreign practitioner who was not qualified to undertake it. An official inquiry is being held; and, meanwhile, the body of the groom has been removed to the Morgue. Till the result be known we refrain from comment, noting merely that the general practice of those countries which, like Switzerland, for instance, only regard doctors as qualified practitioners who hold a Swiss degree, is somewhat a protective measure, the breach of which is rather, except in the eye of the law of course, an economic offence than one within the sphere of medical and general ethics.

### THIS WEEK'S DRUG MARKET.

Business in the Drug market continues to be limited to small transactions, and is, in fact, quieter than is usual at this time of the year. There have been very few changes in value of any importance. Cod-liver oil has been sold at dearer rates, and, as the principal consuming season is approaching, a decline in price is not expected; prices are now at least ten shillings per barrel above the lowest price touched a few months ago. There is still practically no change in the position of opium, the high quotations being well maintained. Damiana leaves are rather dearer. Sarsaparilla has been selling at slightly lower rates. Unfavourable reports of the olive crops have been received from many districts, but from others the reports are favourable; the price of olive oil has been steadily advancing, and the general opinion is that still higher values may be expected.

### TO OUR READERS.

Contributions are specially invited from any of our readers to these columns. They should deal with topical subjects and news. They must be authenticated for the information of the Editor only. The minimum payment if published is 5s. There is no hard-and-fast rule as to space, but notes of about twenty lines in length are preferred.

